# Notice: Pan-American Medical Congress

**Published:** 1893-06

**Authors:** 


NOTICE: PAN-AMERICAN MEDICAL CONGRESS.—The JOURNAL is requested by Prof. H. A. West, M. D., of Galveston, to say that (referring to the notice of the *Pan-American Medical Congress* published elsewhere) those who wish to attend the Congress are requested to notify him, Dr. West, at once, and either send him the registration fee or send it direct to Dr. Owen. As soon as one registers, or notifies Dr. West that he will attend the meeting, Dr. West will issue certificate of delegation.

